# Eco-design for perovskite solar cells to address future waste challenges and recover valuable materials

**DOI:** 10.1016/j.heliyon.2023.e13584

**Published:** 2023-02-09

**Authors:** Elena S. Akulenko, Mahboubeh Hadadian, Annukka Santasalo-Aarnio, Kati Miettunen

**Affiliations:** a Department of Materials Engineering, Faculty of Science and Engineering, 20014 University of Turku, Finland; b Department of Mechanical Engineering, School of Engineering, 11000 Aalto University, Finland

**Keywords:** Eco-design, Photovoltaics, Perovskite solar cells, Material development, Recycling, Circular economy

## Abstract

Photovoltaic development should be steered by the circular economy. However, it is not. In case of perovskite photovoltaics even current environmental directives divert from profitably recycling. Here, we study the profitability of noble metals recovery from wasted perovskite solar cells depending on recycling routes. Our results show that substrates play a major role in the recovery of precious metals and in contrast to previous research even recycling carbon-based devices could reach profitability. Going beyond the recovery of valuable elements, our findings show that revival of the perovskite solar cells is strongly dependent on the device architecture, so far viable for mesoscopic structures with carbon back contacts. Perovskite solar cells are still at the development stage, but the window of opportunity to ensure eco-design will close with market entry, and device complexity might compromise profitability recycling and even result in failure of recovery critical materials. Therefore, its eco-design should be prioritized by materials researchers to develop devices, where valuable components can be separated and liberated with safe and low energy processes.

## 1 Introduction

Perovskite solar cells (PSCs) rival silicon solar cells due to their competitive price, low manufacturing costs, and high efficiency. So far, 25.6% efficiency has been reported for a small-area single-junction cell and 29.15% for a perovskite silicon tandem [1]. PSCs have several architectural options, including flexible cells, which dramatically expand their areas of application [2]. However, PSC technology can only be commercialized after several challenges are resolved such as stability and toxicity [3]. In addition, recycling has been highlighted as a critical challenge [4,5].

PSCs are complex multicomponent devices and are therefore difficult to recycle. So far, several approaches have been proposed albeit only on a laboratory scale. For example, Kadro et al. have considered recycling embedded elements separately, i.e., separating the nanocomponents from conductive glass into pure streams such as Au, I, and Pb, and moderating the potential Pb contamination [6]. Another approach has considered removing the functional layers one-by-one, including both the mechanical and hydrometallurgical steps [[7], [8], [9]]. However, these approaches overlook profitability of recycling. Moreover, there is a lack of understanding about how material selection and the design of PSCs affect recycling. For example, crystalline silicon solar cells, a major source of photovoltaic waste in the coming decade, still lack feasible integrated recycling solutions for treating all bulk and trace materials at a high purity [10]. With other energy technologies, holding a significant market share, such as Li-ion batteries, the complexity of the device requires an energy-intensive multistep process that compromises profitable recycling, and fails to recover critical materials [11]. As discussed in our previous work, recycling takes place if its economically profitable or if its mandated by regulations – best guarantee for ensuring recycling in global scale is profitability [12]. The latter is easier to reach when considering recycling already at the research and design phase, rather than the end-of-life phase. These aspects emphasize the need to direct material research in perovskite cells.

This contribution namely focuses on recycling and how cell design and material options impact its feasibility and profitability. Note that in eco-design of materials recycling is one key issue – usually an overlooked aspect – and other key aspects are efficiency, device lifetime, return on energy investment (impacted also by location), as well as availability and hazardness of materials [12]. Throughout the work we bring up important aspects regarding overall eco-design.

Recycling is a complex issue, which is hindered by a lack of insight. Given this, it has been neglected as a key criterion in materials development. Here lies the main motivation for this work. In this paper, we evaluate the potential for recycling perovskite solar cells and compare the effect of different material and architecture development routes on the recyclability of these energy systems. We explore, in particularly, the options for components which play a key role in profitability of recycling such as substrate and top electrodes. We investigate and identify the optimum economic viability of a potential recycling process based on legislation, business, and materials engineering. We go beyond the recovery of valuable elements and explore the higher levels of recycling such as revival and reuse, which preserves higher product values under less process energy [12]. The four levels of recycling that we investigate are:

1) Revive the device (e.g. reloading perovskite into the cell)
2) Reuse a component (e.g. reutilizing conductive glass)
3) Recycle a material (e.g. recycling PbI_2_)
4) Recover a raw material (e.g. extracting Au or Ag from the cell)

Typically, photovoltaic modules contain Al frames and junction boxes. However, being bulk components, they are easily separated for further processing by mechanical removal. At the moment recycling of Al frame is commercially feasible, and junction boxes are treated as electronic waste [10]. Moreover, frameless modules are lighter and more compact for reverse logistic to recycling site [13]. Therefore, this work focuses the cell level with and without encapsulants.

## 2 Future of degraded perovskite devices

**Fig. 1:**
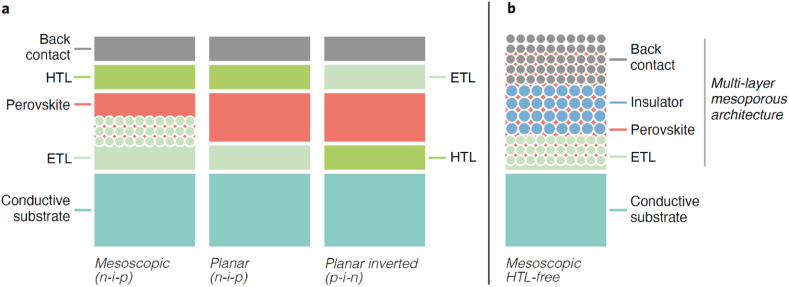
Perovskite solar cells architectures and materials: (a) – common configurations in which the perovskite layer is sandwiched between the charge selective layers as hole transport layer (HTL) and the electron transport layer (ETL). In inverted (p–i–n) structures HTL and ETL are reversed; (b) – mesoscopic structure containing a multi-layer mesoporous architecture such as TiO_2_/ZrO_2_/carbon deposited on conductive substrate in sequence with infiltrated perovskite.

PSCs comprise diverse architectures and materials (Fig. 1) which affect whether the entire device is revived, components are reused, or valuable materials are recovered. From the circular economy perspective, the best strategy is to revive or reuse the entire device to minimize the energy required for recycling and to maximize the value of the products from recycling. If revive or reuse is unfeasible, for example due to component degradation, recycling the components should be considered. The final option is to recover materials at their elemental level. Here, we analyze step-by-step the options for recycling PSCs. Here, we analyze how the pre-commercial perovskite photovoltaics align with eco-design concepts, considering PSCs of common configurations (Fig. 1a) with Au and Ag back contacts and PSCs of mesoscopic structure (Fig. 1b) with carbon back contact. We move from the highest to the lowest levels of processing as reviving device and conclude with recovering elements.

### 2.1 Reviving the product

Although recovered devices may have lower efficiencies, reviving extends the service time of the solar cells [14]. PSCs are hampered by defects in the perovskite layer, which is susceptible to moisture, oxygen and thermal stress. As such, PSCs - can be revived entirely by reloading or regenerating the perovskite material *in situ* and maintaining intact the entire device architecture. This strategy has been employed in devices with a HTL-free mesoscopic structure (Fig. 1b) where the whole mesoscopic scaffold hosts the perovskite absorber [15,16]. The porosity of back contact plays a key role in revival, and it is typically achieved by having a mesoporous carbon electrode [[17], [18], [19]], but alternatively structures based on nanoporous Au [20] and mesoporous Ni [15] electrodes have been demonstrated. Reviving can be achieved in different ways, for instance, through washing degraded perovskite material and reloading it [15,20] or post treating the degraded perovskite material to reconstruct the crystals via the amine gas [16]. If revival of device is based on liquid methods [15,20] then to be feasible, the structure should be resilient enough towards the whole process. For example, when use DMF for carbon-based PSCs, the carbon layer tends to peel off [21].

Unlike the possibility to revive solar cells with HTL-free mesoscopic architectures, the recovery of PSCs, which are fabricated layer-by-layer, is challenging. For instance, in the standard structure in which perovskite is coated by an organic hole selective layer and metal contact (Fig. 1a), it is impossible to avoid severely damaging the upper layers when washing and reloading the perovskite layer. Therefore, besides the structure enabling revival, materials must be suitable for reviving processes, in particular having sufficient stability. For instance, 2,2′,7,7′-tetrakis[N,N-di(4-methoxyphenyl)amino]-9,9′-spirobifluorene (Spiro-OMeTAD) organic hole selective material (Table 1) is not used in this kind of structure as it is challenging to retain the performance of Spiro-OMeTAD in recovered devices. Spiro-OMeTAD deforms easily due to crystallization, photo-oxidation, and ion diffusion.

**Table 1:** Embedded elements costs and amounts. Analysis of embedded elements costs and amounts in PSCs is a starting point to understand the potential motivation (economic value) and hindrances (harmful materials). Au cost was calculated based on gold price 58,3 USD/g29 for 1,9 g/m2 of 100 nm Au layer 6. Ag cost - on silver price 0.8685 USD/g30 for 1.31 g/m2 of 100 nm Ag layer 31. Polyethylene terephthalate covered with conductive Indium Tin Oxide (PET:ITO) cost is provided by Thorlabs [32].

Thickness, μm	Layer	Material	Cost, USD/m^2^	Weight, g/m^2^	Typical device types (structures in Fig. 1)	References
0.1	Back contact	Au	110.77	1.9	n–i–p and p–i–n	[6]
		Ag	1.03	1.31	n–i–p and p–i–n	[31]
5–10		Carbon	<0.3	12.8	n–i–p or HTL-free mesoporous	[[33], [34], [35], [36], [37]]
0.1	Hole transport layer	Spiro-OMeTAD	9.28	0.13	n–i–p	[6,31]
		PEDOT.PSS	0.04	0.15	p–i–n	
0.3–0.4	Light harvesting layer	Metal Halide Perovskite*	1	0.47–1.24**	All types	[6,7,31]
0.025–0.25	Electron transport layer	TiO_2_	0.06	0.53	n–i–p and HTL-free mesoporous	[31,38,39]
		ZnO	0.07	0.7	n–i–p	
		SnO_2_	0.02	0.87	n–i–p	
		PCBM	6.02	0.19	p–i–n	
2000	Conductive substrate	FTO/glass	100	5040	n–i–p and HTL-free mesoporous	[7,37,38]
250		PET/ITO	93.87	200	p–i–n	[12,40]

*The cost and weight were estimated based on MAPbI3.

**0.47 g/m^2^ corresponds to MAPbCl3 layer with density 1.576 g/cm^3^ [41] calculated for 300 nm thickness; 1.24 g/m^2^ corresponds to MAPbI3 layer with density 4.119 g/cm^3^ [41] calculated for 300 nm thickness.

### 2.2 Reusing components

When the entire device cannot be revived, the next option is to reuse the components. Here, individual layers are removed and reused in future solar cells or in other applications. However, reusing the whole layer is challenging because the functional layers (Table 1) are susceptible to damage particularly due to their nanometer-scale thickness, produced by solvent-based or deposition techniques [6]. This is not the case for substrates such as fluorine-doped tin oxide (FTO) glass, which is a major component in emerging solar cells and holds, for example, more than 99% of the total device weight in PSCs (Table 1). Even though FTO glass does not contain rare elements, its reuse is appealing because manufacturing is expensive and energy intensive which means higher emissions of CO_2_ [5]. Moreover, substrate detachment for further reuse would contribute to a large recycling rate-per-mass for the remaining materials. For this reason, the demand for expensive FTO glass will increase drastically in the future regardless of solar-cell technology [22]. This creates a powerful economic incentive for its reuse, which also applies to perovskite solar cells (Table 1). For reuse, the topping electrode layers need to be removed from the transparent conducting substrate: a process that could damage the FTO coating. Demonstrations have confirmed that it is technically possible without causing significant losses to the efficiency of the cell regardless of its structure: planar [23] or mesoscopic [24,25]. In some cases, the recycled substrate demonstrates an even higher efficiency compared to the virgin substrate [26].

Despite the technical viability of reuse, the economics of recycling needs to be investigated further. The reuse of FTO glass currently requires manual labor for disassembly and cleaning. For example, removing the Au contact layers from the substrate with adhesive tape [7] is labor intensive. Reported conductive glass reusing methods use toxic solvents as DMF [27], which have the highest environmental impact [28]. Moreover, FTO glass has been scribed to electrically isolate different sections, which means that devices need to be standardized to reprint new PSCs based on the same shape and geometry. Additionally, there is a lack of industrial-scale processes which can attain virgin quality for recycled FTO. Therefore, especially in the case of the small market share of PSCs, assessments on how large a volume of solar panels would be needed to set up a facility where the FTO glass is drawn apart manually, including the costs for waste shipping to this location.

### 2.3 Recycling compounds

PSCs contain organic and inorganic compounds. Some organic compounds are expensive but cannot be recycled without destroying their structure. For example, Spiro-OMeTAD (Table 1) is an expensive compound, and it would be beneficial to reuse it, but the reported recycling methods employ toxicity solvents, which is less preferable for industrial scale, for example toluene [42]. Even though the trend is to replace the Spiro-OMeTAD, still the state-of-the-art perovskite have the component as giving the highest efficiency [43,44]. In contrast, some inorganic compounds are low cost and abundant, such as TiO_2_, SnO_2_ or ZnO (Table 1). From both the efficiency and stability perspectives, the most promising perovskite structures contain Pb [45]. The existing Life Cycle Assessment (LCA) analysis concludes that the potential environmental impact of Pb-contained PSCs is negligible [37,46,47]. Despite this, there is no sound understanding about the implications of Pb contained PSCs entering the market [48]. To prevent undesirable environmental hazards, these devices should be recycled at their end-of-life and here, the economic profit represents the key driver. Xu et al. reported *in situ* recycling of PbI_2_ [49] and other research groups demonstrated reusing PbI_2_ to prepare perovskite [7,21]. Furthermore, Kadro et al. demonstrated a sequential recycling process for PSCs involving layer-by-layer removal until the FTO-coated glass substrate containing mesoporous TiO_2_ was recovered [9]. These solvent-based methods neutralize Pb cations and prevent them from leaching into the environment. However, the steps to recover the elements from these solvents and to purify the solvents is less studied. A significant drawback of solvent-based methods for perovskite cells is the flammability and toxicity of the solvent. For example, Binek et al. used DMF [7], that would lead to expensive safety-measures on an industrial level. Furthermore, transporting cells from one solvent, then rinsing and placing them with other solvents requires significant processing inputs and therefore, increases overall expenses. However, as this is currently a laboratory-scale solution, further discussion is warranted to establish how perovskite-layer recycling can be scaled up to industrial-scale processing.

### 2.4 Recovering elements

Typically, the recovering of elements is driven by recovery precious metals which help to reach high efficiency [43,44], but their large scale productions might be an issue. Thus, carbon electrodes have been investigated as a replacement for Ag and Au electrodes. That is why further for profitability calculation we consider both options for top electrode: precious metals and carbon.

Despite the proportion of precious metals in e-waste is typically very small, their value can be very high [50]. Moreover, Au and Ag have up to 97% recycling efficiencies for old scrap [51]. To warrant viable recycling, the recovery strategy should be based on the concentration of precious metals used as back contacts in PSCs (Table 1) and their processing methods. For example, Binek et al. demonstrated the manual detachment of nano-width Au contacts from a planar structure using adhesive tape [7]. While a high concentration of Au can be achieved by using manually intensive labor, this would lead to high costs on an industrial scale. Noble metals could potentially be recovered using pyrometallurgy processing, but most likely at the expense of the remaining elements. The advantages of pyroprocessing include the low recycling cost, as manually disassembling or mechanical processing can be avoided. If the cell is treated using hydrometallurgical methods, mechanical processing is needed. Furthermore, the concentration of precious metals needs to be high enough or the scrap must be concentrated before hydroprocessing.

Economically viable amount of Ag and Au for perovskite solar cell recovery are lacking from the literature. Butterman et al. concluded that economically mineable Ag amount is about 700 g/t at 2001 Ag prices [52]. This number needs revision for Ag recovering from e-waste, particular photovoltaics, due to differences in mining and recovering processes as well as the embedded materials and structure of ores and e-waste scrap. Strachala et al. calculated that Ag-containing crushed materials up to 0.07% fails to cover even the cost of basic recycling operations for crystalline silicon panels [53]. Identifying economically viable amount of highly valued Ag and Au is important to develop PSC design for viable recycling.

**Fig. 2:**
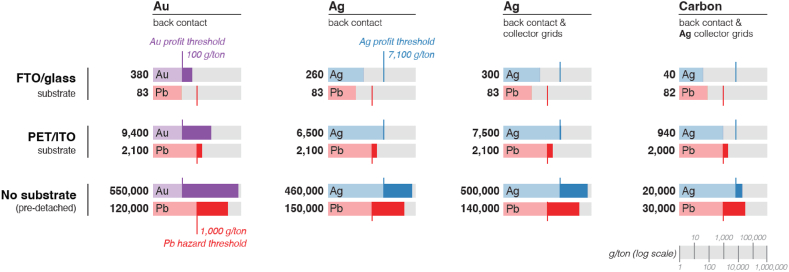
Amount (g/ton) of noble and potentially harmful elements which could be potentially recovered (100% recovery rate) depending on different substrate scenarios. For profitability calculations Spiro-OMeTAD and PCBM were selected as charge transport layers among the expensive ones (Table 1). For substrate pre-detachment scenario (No substrate), elements amounts were calculated based on weight of concentrated waste - the layers left after substrate removal. Noble metals profit thresholds were estimated assuming 80% recovery rate. Pb hazard threshold according to Directive 2011/65/Eu [57], restricting the use of certain hazardous substances in electrical and electronic equipment. The colors indicating the elements (Au – purple, Ag – blue, Pb – red) are marked with dark colors when going above a threshold either in profit or hazard. (For interpretation of the references to color in this figure legend, the reader is referred to the Web version of this article.)

We assume as a preliminary estimation that the retrieval of Au and Ag is economically viable based on the average costs for reverse logistics and processing. As at the time of writing there is no comprehensive data on the economics of PSС recycling, we employ relevant data on the operational expenses (13.5 million USD annually) reported for conventional e-waste recycling plant with a capacity of 2800 tons per year [54] as an inputs in subsequent profitability calculations. Assuming that the extraction rate of metals is 80% [55] and prices are Ag – 0.85 USD/g [30], Au – 58.3 USD/g [29], the amounts needed to cover expenses are 7100 g/ton for Ag or 100 g/ton for Au. Note that these numbers are indicative and will fluctuate based on given market situation, however, they provide insights on the landscape of recovering valuable metals from different devices by pyroprocessing. Depending on the substrate, different revenue can be achieved assuming that the amount of Ag in collector grids in modules is 0.2 g/m^2^ [56]. While the revenue assessment (Fig. 2) shows that glass substrate recycling for Au is already lucrative, this is not the case when recycling Ag, which is unprofitable even when processing collector grids. The recovery of Ag for the PET substrate is still unprofitable. It only begins to cover expenses when additional collector grids connected cells in modules are recycled to increase the total amount of Ag by 15%. Sensitivity analyses shows that the effect of market costs of noble metals provides the highest impact on Au and Ag profitability thresholds. However even under lowest Au costs (35 USD/g) and recovery rate (65%), recovery of Au stays profitable for all three substrate scenarios (Fig. 2), whereas recovery of Ag becomes profitable solely for ‘no substrate’ scenario.

The major difference between PET/PEN and glass substrates arise when aiming to recover elements; plastic-based substrates have a much lower weight compared to glass, and since substrates dominate the overall device weight, major changes in their weight affect the proportion of valuable metals and thus the economics of recycling (Fig. 2). Furthermore, in pyroprocessing for retrieving precious metals, some glass might even benefit the pyroprocess as slag forming agents – limitations exist on how much glass is acceptable. In contrast, PET substrates can be burned resulting in 0.2% [58] of ash and serves as fuel for the process. In fact, if we consider the content of precious metals in the case of PET substrate after pre-burning, the amount of Au increases on 30% and amounts of Ag – on 25% in comparison with the amount for pre-detachment scenario (‘no substrate’). This calculation emphasizes that PET substrate could be favored over glass making even the recovery of Ag highly profitable with preliminary concentrating of waste by burning. Moreover, PET/PEN enables roll-to-roll mass production and employs solar cells in curved surfaces (important for some applications such as vehicles). However, the ability of PET/PEN to protect the active parts of the device from moisture and oxygen can limit the overall lifetime of the device and further protective coatings development might be needed. Typical conducting layer on PET/PEN substrates is ITO, which is an expensive component since it uses rare and expensive In and attempting reuse [59] should therefore account for the limitations of solvent-based recycling (like component reuse for conductive glass as described in Section “Reusing Components”). If that is not possible, In could be recovered in the same pyroprocess with Ag and Au (they can all be covered in the Cu pyroprocess [60]). However, even if In can be recycled, its total reserves are insufficient to support the large-scale manufacturing of photovoltaics to meet global energy needs [59].

Precious metals are not the only options for the back contact. Carbon-based materials are already replacing Au and Ag contacts because of instability in PSCs caused by degradation of the metal contacts and migration of the metal into the perovskite layer [17]. Carbon is an abundantly available and low-cost material. It has facile and low-cost fabrication, a suitable work function, mechanical flexibility, and, most importantly, high stability [19,61]. Although the power conversion efficiency of carbon-based PSCs is lower than Au-based devices [62], carbon electrode-based PSCs without HTL are regarded as a promising alternative architecture to realize low-cost, stable photovoltaics [18]. From an Ag recovering perspective (collector grids), processing carbon-based PSCs is unprofitable (Fig. 2). However, in case of the ‘no substrate’ scenario the amount of Ag in the collector grids which can be recovered arrives at 20,000 g/ton making carbon-based PSCs recycling lucrative. Especially considering that this amount is similar in the case of PET substrate after burning with higher than 5% accuracy.

On panel level devices typically include encapsulation layer. Research on potential commercial encapsulant materials for perovskite modules is ongoing [63,64]. Weight of encapsulant material might vary within the range 138–400 g/m^2^ [65]. Adding an encapsulant of an average weight 270 g/m^2^ will slightly increase the overall weight of -glass based devices, resulting in 5% decreasing in the amount of Ag and Au presented on Fig. 2. At the same time, adding the same encapsulant amount to PET based devices will more than twofold the overall device weight. This decreases the amount of Ag and Au on 57%, making recovery of Ag from PET based devices even more unprofitable. However, encapsulant materials, being organic ones, can be easily burned with PET substrate in pyroprocessing, resulting with 30% increased amount of Au and 25% increased amount of Ag in comparison with ‘no substrate’ scenario (Fig. 2).

### 2.5 Harmful elements interfering valuable elements recovery

The PSCs contains potentially harmful elements (Pb and halogens), which can interfere or prevent the recycling of critical materials such as Ag and Au.

**Fig. 3:**
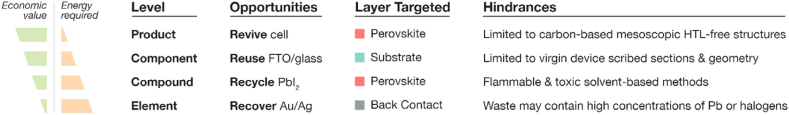
Levels of recycling for perovskite solar technology. Higher levels of recycling preserve economic value. If reviving the perovskite solar cells fails for several reasons, including the end-of-life of the perovskite layer, the photovoltaic devices can be recycled to reuse the valuable components by disassembling and reassembling the layers. If the compounds are expensive, scarce, or harmful, the incentive to recycle increases. When recovering elements is the only option, the main economic incentive originates from precious metals.

If Au and Ag are the only economically valuable elements, these cells could be directed to pyrometallurgical high-temperature processes where all other elements would potentially be lost. For example, when we consider the ‘no substrate’ scenario, allowing the substrate to be reused, the proportion of precious metals in the remaining layers increases significantly (Fig. 2). However, this scenario also results in >120,000 g/ton of Pb (Fig. 2), and therefore challenges the use of pyroprocessing (Fig. 3). Here, a key concern is to safely recover Pb containing species. As previously mentioned, the perovskite layer cannot be removed in a single process step. So far on a laboratory scale, multistep solvent-based methods have been developed to liberate perovskite constituted elements for further processing and/or recycling. However, application to large-scale recycling is missing from the literature.

Another aspect that should be considered is legislation. According to Directive 2011/65/Eu [57] the maximum concentration of Pb tolerated by weight in homogeneous materials is 0.1%. This requirement is pushing towards smaller concentrations. Reducing the amount of Pb per area is challenging. As such, smaller concentrations can be achieved by making the device thicker. In addition, to comply with the criteria for a homogenous material, the different layers should be inseparable. Making the device thicker will not only dilute the amount of Pb but also the other valuable materials such as Ag and Au reducing the economic profitability of element recovery. Furthermore, from recycling perspectives it would be important to develop structures that can be easily separated (heterogenous) – another major conflict between commercializing and recycling. It is worth noting that the Directive relates only to the consumer market but not to large-scale fixed installations such as, for example, solar farms. To maximize the impact of commercializing and recycling PSCs large-scale installations, for example solar farms or solar building-integrated panels, should be investigated in more detail.

Concerning halogens, as in the case of Pb halide, a small amount causes no environmental risks during the recycling process. In contrast, substantial amounts received after substrate detachment (Fig. 2) can incur additional cleaning-equipment costs or in the worst-case scenario, acid rain if halogens are released into nature through exhaust fumes. This could be resolved if non-halogen materials could be incorporated into the design of the device, assuming that a change in the material would not impact negatively the efficiency or stability of the system.

Nonhalide X-site anions in perovskite materials have been developed to overcome stability issues caused by the specific ionic character of the metal-halide bonds due to the high electronegativities of halogen atoms [66]. The alternatives for halide ions in the perovskites include monovalent and divalent anions.

A few suitably sized monovalent anions exist that fulfill the Goldschmidt tolerance factor (used for the stability and distortion of crystal structures in perovskites). However, some pseudohalides with a similar spatial size and characteristics as halides, such as thiocyanate ion (SCN^−^) and formate (HCOO^−^), have been employed in perovskites both as additives and alternatives to replace the halides [67]. The thiocyanate anion has been incorporated into the perovskite structure to form (A_2_B(SCN)_2_X_2_) [66]. Nevertheless, the wide bandgap and constricted carriers in perovskites with layered structures limit the development of solar cells with high efficiency. More importantly, these cells are also vulnerable to humidity, which presents challenges during the recycling process [68].

In perovskite structures, divalent anions, particularly chalcogenide anions, have emerged as potential alternatives for halide anions partially or fully. Due to the covalent bonding between the transition metals and the chalcogenides, the stability of the perovskite can be improved. Metal chalcogenides, proposed as potential solar cell materials in 2015 [69], have exhibited bandgaps suitable for photovoltaic applications. The use of divalent anions in perovskite requires trivalent or tetravalent B cations to maintain charge neutrality in the structure [66]. If Pb is replaced by a less toxic cation based on the Group-IIA (Ca, Sr, and Ba) and Group-IVB (Ti, Zr, and Hf) elements, which are more environment-friendly [69], this could have a beneficial impact. Computational studies propose a series of chalcogenide perovskites that exhibit a suitable bandgap, strong absorption coefficient, and low effective masses of charge carriers [70,71]. Many chalcogenide perovskites such as BaZrS_3_, CaZrS_3_, SrTiS_3_, and SrZrS_3_ have been experimentally synthesized. However, synthesizing and processing chalcogenide perovskites is difficult compared to halide perovskites, and the solar cells from this class of perovskite have rarely been reported [72,73].

## 3 Conclusions

This work discusses the future of degraded PSCs at the junction of materials engineering, business, and legislation. Perovskite cells require special treatment at their end-of-life because they contain toxic Pb. At the same time, recovering rare expensive metals like Au and Ag from back contacts is important to prevent a shortage in supply in the coming decades. We discuss recycling options for PSCs based on various levels of recycling from reviving devices to recovering elements. However, the feasibility of recycling is limited by several technical challenges such as solar-cell architecture, degradation mechanisms and intensive manual labor. For instance, for the highest recycling level - revival of the PSC - the porosity of back contact plays a key role, and it is typically achieved by having a mesoporous carbon electrode. Another example is the reuse of conductive glass substrate where despite its technical feasibility, profitability is limited by the geometry of the virgin device and the intensive manual labor. Furthermore, a greater understanding is needed about the feasibility of recycling on an industrial scale, particularly regarding applied toxic solvents such as DMF.

The lowest recycling level - recovery of precious metals - is influenced by the substrate type and the proportion of harmful components in the treated waste. At the time of writing, it is economically viable to retrieve Au (>100 g/ton) from FTO glass-based devices. On the contrary, Ag back contacts fail to yield economically profitable amounts (>7100 g/ton) from devices prepared on both conductive glass and PET. Ag reaches profitability in case of “no substrate” scenario or PET substrate after burning. The latter results in 25% increased amount of Ag in comparison with ‘no substrate’ scenario. In contrast to established opinion, our study shows that the recycling of carbon-based PSCs can reach viability in case of ‘no substrate’ scenario by recovering 20,000 g/ton collector grid Ag. This amount is similar in the case of PET substrate after burning with higher than 5% accuracy which can be attractive for recyclers. As precious metal prices are volatile, we recommend recalculating the economic viability of recovering Ag and Au based on real-time prices.

Regarding harmful elements, further investigation is warranted. According to our findings, the ‘no substrate’ scenario increased the proportion of precious metals and allowed the substrate to be reused. However, this came at the expense of >120, 000 g of Pb per ton of processed waste, which increases the environmental risks of pyroprocessing for non-diluted substrate-free waste streams. In addition, legislation imposes restrictions on the levels of Pb in consumer products. This may cause a conflict of interest in the future between the manufacturers and the recyclers of perovskite cells, limiting the market to large-scale installations such as solar farms or building integrated panels. Further research is needed in this direction to identify a compromise. Since PSC technologies are still at the research and development stage, different recycling strategies should already be considered when developing new materials and designs for photovoltaic devices.

## 4 Method details

### 4.1 Circular economy and eco-design concepts

In this work we use the method proposed in our previous article based on circular economy concept and eco-design approach to energy systems development [12]. The method helps to evaluate recyclability both individual components and whole PV system, considering recycling process as consisting of four levels defining by energy inputs and product value (Fig. 3):

• reviving devices, i.e. restoring performance of PV system;
• reuse components, i.e. reutilizing the components for the further use in the same or other system;
• recycle compounds, i.e. separating the compounds the further use in the same or other system;
• recovery valuable elements, i.e. extracting elements from PV waste.

### 4.2 Data

#### 4.2.1 Existing recycling plant data

We assume the potential parameters and operational costs of average perovskite PV recycling plant from the e-waste recycling profitability study conducted by Lappeenranta University of Technology [54], which calculate operational costs (collection and processing) and treatment capacity of an average e-waste plant based on the data analyzed for reported conventional e-waste recycling plants in United States, Great Britain, China. The following inputs were used for calculation of amount of Ag, Au and Pb after pyroprocessing of wasted perovskite PVs:

• treatment capacity – 2800 tons of e-waste per year;
• collection costs – 1970 USD per ton of e-waste;
• processing costs – 2850 USD per ton of e-waste.

Operational costs were assumed as 13,5 million USD annually based on Eq. (1):

(1) $$2,800\cdot \frac{\left(1970+2850\right)}{10^{6}}=13,5$$

In our study for different scenarios we assume under e-waste: non-diluted wasted perovskite cells with and without Ag collector grids; preliminary concentrated waste of perovskite cells by substrate detachment or pre-burning step (“no substrate” scenario).

### 4.3 Embedded materials and cell architecture data

For analysis of fist three levels recycling in view of eco-design concept:

• reviving devices;
• reusing components;
• recycling compounds,

we considered PSCs of common configurations (Fig. 1a, Table 1) with Au and Ag back contacts and PSCs of mesoscopic structure (Fig. 1b, Table 1) with carbon back contact.

For analysis of the last level:

• recovering valuable elements,

we performed economic assessment by conducting our own calculation based on the materials weight and costs presented in Table 1. For these profitability calculations we consider only common planar architecture where we changed only back contact materials (Au, Ag and carbon) and substrate materials (conductive glass and PET). Other functional layers as charge transport layers (Spiro-OMeTAD, PEDOT. PSS) and the most commonly used perovskite MAPbI3 [44] were assumed to be the same, because they have neglectable impact on calculations of precious metals due to its small quantities. For carbon back contact additionally impact of Ag collector grids was assessed for “no substrate” scenario assuming that the amount of Ag in collector grids in modules is 0.2 g/m^2^ [56]. The data in Table 1 were collected from publicly available sources except cost of Au and Ag in USD/m^2^. These values were calculated by our own based on the Au an Ag prices actual on the date of calculation [29,30].

Amount (g/ton) of Ag, Au and Pb which could be potentially recovered (100% recovery rate) depending on different substrate scenarios (Fig. 2) were calculated by means of Eq. (2):

(2) $$A_{n}=\frac{10^{6}}{w_{total}}w_{n}$$

wherein

• A_n_ – amount (g/ton) of Au, Ag or Pb which could be potentially recovered (100% recovery rate);
• W_total_ – total weight of cell in g/m^2^;
• W_n_ – weight of embedded element/compound in g/m^2^.

Profitability thresholds for Au (100 g/ton) and Ag (7100 g/ton) were estimated by means of Eq. (3):

(3) $$A_{profit}=\frac{Annual\;operational\;costs}{\left(Annual\;treatment\;capacity\cdot Recovery\;rate\cdot Cost\;of\;noble\;element\right)}$$

wherein

• Annual operational costs - 13.5 million USD;
• Annual treatment capacity - 2800 tons per year;
• Recovery rate – 80% [55]
• Cost of Ag – 0.85 USD/g [30], Au – 58.3 USD/g [29].

### 4.4 Quantification and statistical analysis

**Fig. 4:**
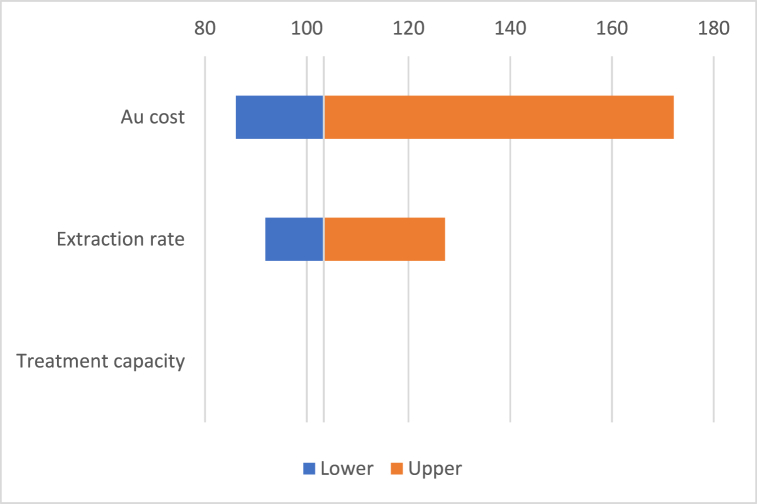
Au profitability sensitivity.

**Fig. 5:**
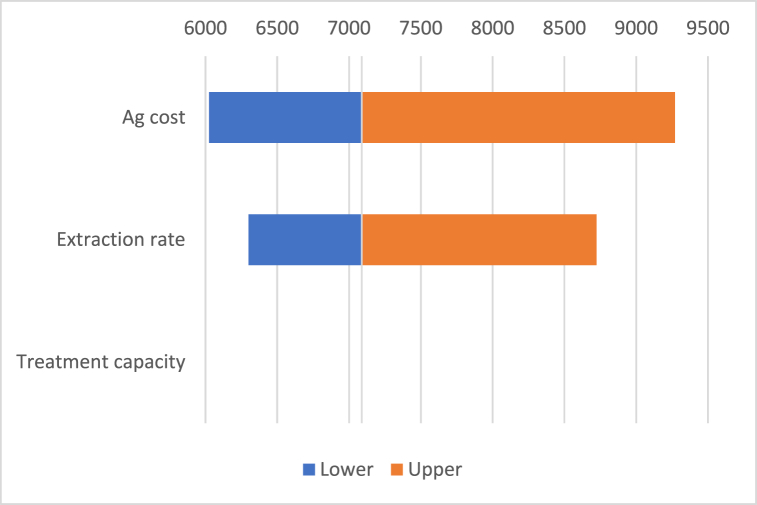
Ag profitability sensitivity.

Sensitivity analyses have been conducted to examine the influence of changes in value of noble metals profitability thresholds (Au – 100 g/ton, Ag – 7100 g/ton). Taking into consideration that the cost of noble metals are ones of the main variables to decrease the profitability of perovskite solar cells recycling, we conducted a sensitivity analysis for a range of Au cost from 35 to 70 USD/g, a range of Ag cost from 0.65 to 1 USD/g depending on a range of annual treatment capacity of recycling plant from 2500 to 3000 ton of waste per year and recovery rate from 65% to 90% (Fig. 4, Fig. 5). The sensitivity of the results on annual treatment capacity is relatively low. For example, decreasing the treatment capacity to 2500 ton per year is directly proportional to the decreasing in annual processing costs (12.1 million USD). That means that the Au and Ag profitability thresholds remain same for the range of treatment capacity from 2500 to 3000 ton of waste per year considering cost and recovery rates of these metals as constant variables. Noble metals profitability thresholds are moderately sensitive to recovery rates. When recovery rate decreases from 80% to 65%, the profitability thresholds increase for Au to 130 g/ton and for Ag to 8700 g/ton. The effect of market cost fluctuation of these metals provides the highest impact on profitability thresholds. For example, decreasing in Au cost to 35 USD/g results in decreasing of profitability Au thresholds to 170 g/ton, decreasing in Ag cost to 0.65 USD/g results in decreasing of profitability Ag thresholds to 9300 g/ton. However even under lowest Au costs and recovery rates, recovery of Au stays profitable for all three substrate scenarios (Fig. 2), and recovery of Ag would be profitable solely for ‘no substrate’ scenario.

Au profitability threshold (100 g/ton) depending on Au cost, which ranges from 35 to 70 USD/g treatment capacity of recycling plant, which ranges from 2500 to 3000 ton of waste per year, and recovery rate, which ranges from 65% to 90%.

Ag profitability threshold (7100 g/ton) depending on Ag cost, which ranges from 0.65 to 1 USD/g treatment capacity of recycling plant, which ranges from 2500 to 3000 ton of waste per year, and recovery rate, which ranges from 65% to 90%.

## Author contribution statement

Elena S. Akulenko: Performed the experiments; Conceived and designed the experiments, Analyzed and interpreted the data, Contributed reagents, materials, analysis tools or data, Wrote the paper.

Mahboubeh Hadadian, Annukka Santasalo-Aarnio, Kati Miettunen: Conceived and designed the experiments, Analyzed and interpreted the data, Contributed reagents, materials, analysis tools or data, Wrote the paper.

## Funding statement

Kati Miettunen was supported by 10.13039/501100002341Academy of Finland [336577 & 336441].

Annukka Santasalo-Arnio was supported by 10.13039/501100002341Academy of Finland [326346].

Elena S. Akulenko was supported by Fortum and Neste Foundation [20210154].

## Data availability statement

Data included in article/supp. material/referenced in article.

## Declaration of interest's statement

The authors declare no competing interests.
